# NK cells in peripheral blood carry trogocytosed tumor antigens from solid cancer cells

**DOI:** 10.3389/fimmu.2023.1199594

**Published:** 2023-08-01

**Authors:** Mauricio Campos-Mora, William Jacot, Genevieve Garcin, Marie-Lise Depondt, Michael Constantinides, Catherine Alexia, Martin Villalba

**Affiliations:** ^1^ IRMB, Univ Montpellier, INSERM, Montpellier, France; ^2^ Institut du Cancer de Montpellier (ICM) Val d’Aurelle, Montpellier University, INSERM U1194, Montpellier, France; ^3^ IRMB, University of Montpellier, INSERM, CNRS, Montpellier, France; ^4^ Institut du Cancer Avignon-Provence Sainte Catherine, Avignon, France

**Keywords:** NK cells, t-SNE, trogocytosis, breast cancer, CD45

## Abstract

The innate immune lymphocyte lineage natural killer (NK) cell infiltrates tumor environment where it can recognize and eliminate tumor cells. NK cell tumor infiltration is linked to patient prognosis. However, it is unknown if some of these antitumor NK cells leave the tumor environment. In blood-borne cancers, NK cells that have interacted with leukemic cells are recognized by the co-expression of two CD45 isoforms (CD45RARO cells) and/or the plasma membrane presence of tumor antigens (Ag), which NK cells acquire by trogocytosis. We evaluated solid tumor Ag uptake by trogocytosis on NK cells by performing co-cultures *in vitro*. We analyzed NK population subsets by unsupervised dimensional reduction techniques in blood samples from breast tumor (BC) patients and healthy donors (HD). We confirmed that NK cells perform trogocytosis from solid cancer cells *in vitro*. The extent of trogocytosis depends on the target cell and the antigen, but not on the amount of Ag expressed by the target cell or the sensitivity to NK cell killing. We identified by FlowSOM (Self-Organizing Maps) several NK cell clusters differentially abundant between BC patients and HD, including anti-tumor NK subsets with phenotype CD45RARO+CD107a+. These analyses showed that *bona-fide* NK cells that have degranulated were increased in patients and, additionally, these NK cells exhibit trogocytosis of solid tumor Ag on their surface. However, the frequency of NK cells that have trogocytosed is very low and much lower than that found in hematological cancer patients, suggesting that the number of NK cells that exit the tumor environment is scarce. To our knowledge, this is the first report describing the presence of solid tumor markers on circulating NK subsets from breast tumor patients. This NK cell immune profiling could lead to generate novel strategies to complement established therapies for BC patients or to the use of peripheral blood NK cells in the theranostic of solid cancer patients after treatment.

## Graphical Abstract

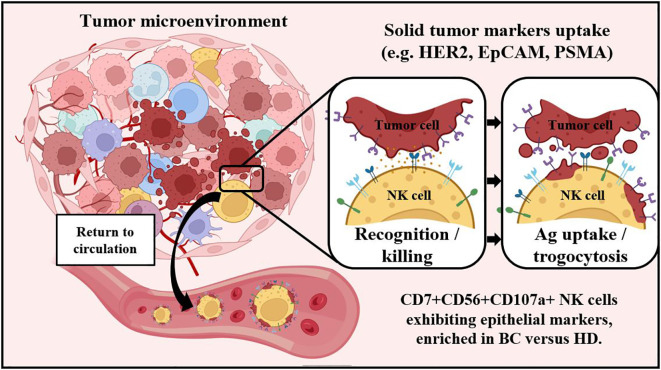
Human NK cells that encounter cancerous cells uptake epithelial antigens (Ag) and can be identified on blood of breast tumor patients comprising specific NK subsets.

## Introduction

Natural killer (NK) cells are a subset of lymphoid cells and part of the innate immune compartment. As blood-circulating cells with cytotoxic activity, NK cells screen for damaged or stressed cells, and they are readily able to kill virus-infected or transformed tumor cells, contributing to immune surveillance (1). These cells are mainly classified as CD56+CD3- innate lymphoid cells, but they constitute a heterogeneous population comprising NK cell subsets with different cytotoxic potential. Based on CD56 and CD16 surface expression, they subdivide into CD56^+^CD16^high^ blood-circulating NK cells, with stronger cytotoxic activity after target cell recognition, and CD56^high^CD16^low^ cells cytokine-producing NK cells with poor cytolytic activity, mostly present in secondary lymphoid tissues (2). However, recent studies have challenged this classical view, and high dimensionality, single-cell proteomic analysis have revealed a striking NK cell phenotype diversity, which might be influenced by both genetic differences between individual humans and environmental conditions (3). Considering that NK cell function is tightly modulated by the expression of several inhibitory and activating receptors, the determination of NK cell subsets repertoire and their contribution to physiological processes could be of paramount importance for generation of NK cell-based therapies against malignant diseases (4, 5).

The anti-tumor properties of NK cells have been previously discussed (6). NK cells play an essential role in tumor clearance by recognizing and killing abnormal tumor cells without the need of prior activation. After recognition of target cells, different target cell-derived proteins can be acquired in NK cell membrane surface in a cell-to-cell contact-dependent manner, a process called trogocytosis (7–11). Trogocytosis involves an intercellular transfer of membrane patches, and it has been shown to occur in different immune cell types, albeit the physiological relevance of this process is not fully understood (9, 11–13). This transference of functional proteins to cell surface could modulate NK function *in vitro* and *in vivo* (14–20). Trogocytosis is receiving high interest from the clinic for this possibility to modulate NK cell (18, 20) or CAR T cell function (21, 22).

We have reported the identification of NK cell populations with anti-tumor activity in hematological cancer patients (23–26). The highly activated CD56+CD16^high^ NK cells found in these patients exhibit the expression of activation markers, such as NKp46 and NKG2D, and low expression levels of inhibitory markers, such as NKG2A and CD94. Interestingly, these NK cells also present non-NK, tumor cell-derived antigens on their surface, which can be an indicative of trogocytosis during cell killing (23–26). We found that this subset of anti-tumor NK cells is also characterized by degranulation and co-expression of both CD45RO and CD45RA (CD45RARO cells) (23–26). Further high-dimensionality, multiparametric flow cytometry and unsupervised analyses in multiple hematological tumor patients showed that NK subsets presenting CD45RARO phenotype and evident tumor-antigen derived trogocytosis responds directly to the oncologic status of patients, which suggest that the frequency of the function of these NK subsets depend of the presence of targets (23, 24).

NK cells infiltrate solid cancers, as well as tumor-infiltrated lymph nodes and metastases (27–29). NK cell infiltration of most solid tumor is rather sparse and depends on tumor localization and the nature of the cancer (30). To our knowledge, detection of anti-tumor NK cell subsets expressing trogocytosed tumor-derived markers in peripheral blood of solid tumor patients have not been reported. Here we investigated whether NK cells could acquire solid tumor antigens by trogocytosis *in vitro* and *in vivo*. Additionally, we studied the presence of anti-tumor, blood-circulating, NK cells subsets exhibiting these solid tumor antigens in breast cancer patients by multiparametric flow cytometry and high-dimensionality unsupervised analyses.

## Patients, materials and methods

### Ethical statement

The use of human specimens for scientific purposes was approved by the French National Ethics Committee. All methods were carried out in accordance with the approved guidelines and regulations of this committee. Written informed consent was obtained from each patient or donor prior to collection.

### Breast tumors patients

Data and samples from patients were collected at the Institute for Cancerology of Montpellier (ICM), France, after patient’s written consent and following French regulations. Patients were enrolled in the ICM-BDD 2017/37 (ID-RCB: 2017-A01940-53) clinical program approved by the “Comités de Protection des Personnes Sud-Ouest et Outre-Mer III” with the reference 2017/45. Blood samples were collected at diagnosis. Peripheral blood mononuclear cells (PBMCs) were obtained by Ficoll® gradient and stored frozen in liquid nitrogen until use. Patients’ status is described in **Table 1**.

**Table 1:** Description of patients.

Patient Number	Pathology	Estrogen Receptor (%)	Progesterone Receptor (%)	HER2 Overexpression	Patients ER/PR Receptor status	Clinical Status
1	Breast cancer	0	0	3+	–	Alive
2	Breast cancer	100	70	2+/FISH-	+	Alive
3	Breast cancer	100	100	1+	+	Alive
4	Breast cancer	100	70	0	+	Alive
5	Breast cancer	90	90	1+	+	Alive
6	Breast cancer	60	30	1+	–	Alive
7	*In situ* breast cancer carcinoma	ND	ND	ND	–	Alive
8	Breast cancer	95	5	2+/FISH-	+	Alive
9	Atypical ductal hyperplasia	ND	ND	ND	–	Alive
10	Breast cancer	100	5	2+/FISH-	+	Alive

Blood samples were collected from patients and PBMCs were frozen until use. The clinical status of the patients is depicted regarding expression of estrogen and progesterone receptors and HER2 status (0, 1+, 2+/FISH- are, altogether “HER2-negative”, and 2+/FISH+ and 3+ are “HER2-positive”. Patients 7 and 9 were included in this study, but were not be considered per se cancers. Patient 7 developed an *in situ* carcinoma and consequently ER, PR and HER2 were not tested. Patient 9 showed atypical ductal hyperplasia at recruitment, indicative of an increased risk of cancer, and, indeed, the patient developed invasive cancer the following year. ER, PR and HER2 are typically not tested in this population. The column “Patient Receptor status” describes the patients who are considered in the group of receptor-negative patients (–) or receptor-positive patients (+).

FISH, fluorescence *in situ* hybridization; ER, estrogen receptor; HER2, human epidermal growth receptor 2; ND, not determined; PR, progesterone receptor.

### Healthy donor

HD samples were obtained from written informed donors, collected by clinicians of the CHU Montpellier and collected and processed as the patient’s samples.

### Cell lines

Breast cancer cell lines BT-20, SKBR3 and MDA-MB-468, pancreatic adenocarcinoma cell line LNCaP, colorectal adenocarcinoma cell line HCT116, and the Epstein-Barr Virus (EBV)-transformed lymphoblastoid B cell line PLH were grown in RPMI 1640 media (Gibco) supplemented with 10% fetal bovine serum (FBS). Cells were used for experiments at confluency of ~80%. Cell line identity was confirmed by flow cytometry when possible, and cells were regularly tested for mycoplasma.

### UCBMC purification

Umbilical cord blood (UCB) units obtained from healthy donors from CHU Montpellier. UCB mononuclear cells (UCBMC) were collected from UCB units using Ficoll® Paque Plus (Sigma) by density gradient centrifugation. Briefly, one volume of Ficoll® Paque Plus were added to conical tubes, and two volumes of blood (previously diluted 1:1 with RPMI media) were slowly deposited at the top. Tubes were centrifuged at 425 x *g* for 30 min at room temperature, without brake. Mononuclear cells were collected from buffy coat layer, washed in RPMI and resuspended in RPMI media supplemented with 10% FBS.

### Enrichment, activation and expansion of human NK cells

Expanded NK (eNK) cells were obtained as previously described (31–34). Briefly, UCBMC were depleted of T cells by using EasySep™ CD3 Positive Selection Kit II (STEMCELL Technologies). Cells were cultured in the presence of γ-irradiated PLH cells at ratio NK-to-accessory cell of 1:1 in RPMI 10% FBS media supplemented with human IL-2 (100UI/mL, Peprotech) and human IL-15 (5 ng/mL, Miltenyi Biotec) for 14-to-21 days. Once every 3 days, cells were counted and fresh culture media with FBS, IL-2 and IL-15 was added to the culture, along with additional γ-irradiated PLH cells. Purity of human CD3-CD56+ eNK cells at the end of the culture was always ≥ 90%.

### Trogocytosis *in vitro* and staining

Adherent tumor cells were seeded in 48-well flat-bottom plates (200,000 cells/mL in culture medium) and incubated overnight at 37°C. Trogocytosis *in vitro* of tumor markers was attained by co-culturing eNK cells (acceptor cells) with BT20, LNCaP, SKBR3, MDA-MB-468 or HCT116 (donor cells) in several acceptor-to-donor ratios. For experiments under inhibition of actin recruitment, eNK cells were previously treated with 2 µg/mL cytochalasin D (CytD, Sigma-Aldrich) for 30 min at 37°C. In experiments where Src kinase was inhibited, NK cells were pre-treated with 10 µM PP2 (Sigma) for 10 min and then maintained during the co-culture. NK cells were also co-cultured with tumor cells at 4°C as a control. For membrane dye transfer experiments, SKBR3 or MDA-MB-468 cells were stained with Vybrant™ DiD Cell-Labeling Solution (Invitrogen) for 20 min, and then co-cultured with NK cells. Plates were centrifuged at 150 x *g* for 30 seconds to favor cell contact, and cells were incubated at 37°C for 2 h or overnight (16 h). After trogocytosis, eNK cells were recovered and washed several times in FACS buffer (PBS, 2% FBS). For experiments to measure tumor marker trogocytosis, cells were stained for surface markers with the following fluorochrome-coupled antibodies: CD56-V450; CD19-ECD; HER2-PE (all from BD); CD16-KO (Beckman); CD3-APC; CD335-PE-Vio770; CD45RO-FITC; CD45RA-APC-Vio770; and PSMA-PE or EpCAM-PE (all from Miltenyi Biotec). For experiments to determine measuring membrane dye transfer, cells were for surface markers with the antibodies CD56-V450 and CD3-FITC (all from BD). Cell viability was determined using 7-AAD exclusion (Miltenyi Biotec). Staining was performed in FACS buffer at 4°C for 25-30 min, and then cells were washed three times before FACS acquisition and analysis in Gallios flow cytometer instrument (Beckman Coulter). After exclusion of doublets and dead cells, tumor marker detection on CD3-CD56+CD335+ NK cells was evaluated by analyzing FCS files using FlowJo software v10.6.1 (Tree Star Inc.).

### Trogocytosis *ex vivo*

In brief, healthy donors and patient’s total peripheral blood mononuclear cells (PBMC) were stained for surface markers with the following fluorochrome conjugated antibodies: CD4-BUV737, CD56-BUV395, CD3-BV786, CD16-BV711, CD7-BV421, HER2-BV650, CD326-BV605, PD1-FITC, MUC1-PerCP, CD107a-PE-CF594, CD14-AF700, CD19-AF700, PSMA-PE, CD45RO-VioGreen, NKp46-PE-Vio770, CD45RA-APC-Vio770 and CD138-APC (all from Miltenyi Biotec). Cell viability was determined using DAPI exclusion (BD Biosciences). Cells were stained with antibodies cocktail in FACS buffer at 4°C for 25-30 min and washed twice with the same FACS buffer and acquired on BD LSR-Fortessa instrument (Blue-Yellow/Green-Red-Violet-Ultraviolet) (BD Bioscience). FCS files were analyzed using FlowJo software v10.6.1 (Tree Star Inc.).

### Fluorescence microscopy

Breast cancer cell line SKBR3 cells were seeded in 24-well flat-bottom plates (200,000 cells/mL in culture medium) on coverslips previously coated with poly-D-lysine (Sigma), and incubated overnight at 37°C. Trogocytosis *in vitro* of tumor markers was attained by co-culturing eNK cells (acceptor cells) with SKBR3 (donor cells) in effector-to-target ratio of 1:1, leaving some coverslips with eNK alone. Cells were incubated overnight at 37°C and after media removal and wash with PBS, attached cells were fixed using 4% paraformaldehyde diluted in PBS and blocked with 5% FBS diluted in PBS. Cells were then incubated with CD56-AlexaFluor™488 and CD326-PE antibodies (all from BD Biosciences) diluted in blocking solution for 2 h at room temperature, protected from light. Some coverslips with eNK cells were stained with CD45-PE antibody (Beckman) diluted in blocking solution. Hoescht 33342 was used for nucleus staining. After several washing steps, coverslips were mounted on microscope glass slides using Prolong Gold mounting media (ThermoFisher). Cell samples were visualized using a Leica SP5 fluorescence microscope (Carl Zeiss, Germany) and images analyzed using the Las X Life Science software (Carl Zeiss).

### High dimensional reduction analysis

To generate tSNE or UMAP embedding, a pre-gated NK cell population from each sample with the same number of cells per patient and timepoint was selected using FlowJo Downsample plugin (v3.1.0) and merged before uploading in the Cytobank cloud-based platform (Cytobank, Inc.). High-dimensional single-cell data dimensionality reduction was performed by viSNE, which is based upon the t-Distributed Stochastic Neighbor Embedding (t-SNE) implementation of Barnes-Hut (35). viSNE was used to visualize FACS data as 2D t-SNE maps, using the following parameters: Desired Total Events (Equal sub-sampling): 50.000; Channels: selected all 16 surface markers; Compensation: “File-Internal Compensation”; Iterations: 1000; Seed; “Random”; Theta: 0.5. FlowSOM was used with default settings unless otherwise noted. FlowSOM uses Self-Organizing Maps (SOMs), based on marker expression phenotype, to assign all individual cells into clusters and metaclusters (that is, group of clusters) (36). FlowSOM was performed with the following parameters: Event Sampling Method: “Equal”; Desired events per file: “2.736”; Total events actually sampled: “27.160”; SOM Creation: “Create a new SOM”; Clustering Method: “Hierarchical Consensus”; Number of metaclusters: “12”; Number clusters: “256”; Iterations: “100”; Seed: “4567”. CITRUS (cluster identification, characterization, and regression) is an algorithm designed for the discovery of statistically significant stratifying biological signatures within single cell datasets containing numerous samples across associated conditions or correlated with clinical phenotype of interest (e.g. responders versus non-responders) (37). The output is a network topology of cell subpopulations divided in sub-clusters that represents a hierarchical stratification of the original sample. Median expression levels of functional markers measured across each population can drive the differentiation between phenotypes. CITRUS was performed using the Significance Analysis of Microarrays (SAM) correlative association model (Benjamin-Hochberg-corrected *P* value, false discovery rate (FDR) < 0.01), with the following parameters: Clustering channels: “selected all surface markers except CD107a and tumor markers”; Compensation: “File-Internal Compensation”; Statistic channels: “CD107a and tumor markers”; Association Models: “Significance Analysis of Microarrays (SAM) – Correlative”; Cluster Characterization: “Medians”; Event sampling: “Equal”; Event sampled per file: “2000”; Minimum cluster size (%): “1”; Cross Validation Folds: “5”; False Discovey Rate (%): “1”. Identification of NK cell subsets between the group “Healthy Donors” and “Breast Cancer patients” was performed by comparing the relative expression of CD107a and tumor markers of the specified FlowSOM metacluster.

### Statistical analysis

Experimental figures and statistical analysis were performed using GraphPad Prism (v8.0). All statistical values are presented as * p<0.05; ** p<0.01; *** p<0.001 and **** p<0.0001. Mean values are expressed as mean plus or minus the standard error of the mean (SEM).

## Results

### NK cells perform trogocytosis *in vitro* on solid tumor cells

The breast cancer cell lines BT-20 and SKBR3, the human prostate adenocarcinoma cell line LNCaP and the human colon cancer cell line HCT116 lack expression of CD3 (T cell marker) and CD56 (NK cell marker) and show very low expression of CD335 (NK cell marker), also known as NKp46/NCR1 (**Figure S1**). In contrast, they do express human epidermal growth factor receptor 2 (HER2) and Epithelial cell adhesion molecule (EpCAM or CD326).

We expanded NK cells (eNK) *in vitro* as previously described (31–34) and incubated them with BT-20, SKBR3 and LNCaP cell lines. All these cell lines were sensitive to eNK cell killing (**Figures S2A, B**).

**Figure 1:**
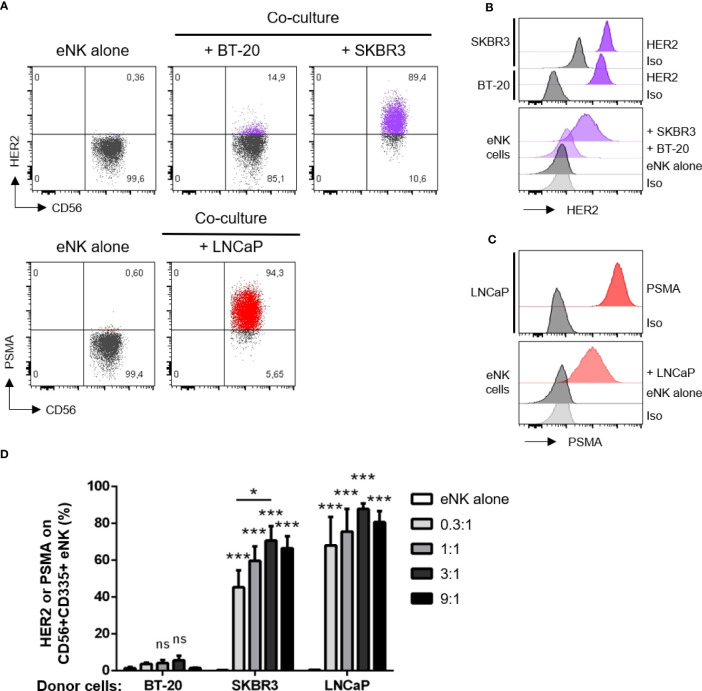
NK cells acquire HER2 and PSMA from solid tumor cells. NK cells expanded *in vitro* (eNK) were incubated overnight with different solid tumor cells. After excluding doublets and dead cells, the surface expression of HER2 (for co-culture with BT-20 or SKBR3 cells) and PSMA (for LNCaP cells) were analyzed on live CD56+CD335+ eNK cells by flow cytometry. **(A)** Expression of HER2 or PSMA on eNK incubated alone or co-cultured at E:T 3:1 with the depicted cell lines. **(B)** Top, histograms show HER2 expression on SKBR3 and BT-20 cell lines. Bottom, HER2 expression level on eNK cells alone or co-cultured at E:T 3:1 with tumor cells. **(C)** Top, histograms show PSMA expression on LNCaP cell line. Bottom, PSMA expression level on eNK cells alone or co-cultured at E:T 3:1 with LNCaP cells. **(D)** The percentage of eNK carrying HER2 or PSMA is depicted in the graph (bars represent mean ± SEM). Statistical significance between eNK alone condition and the different E:T ratios were determined by two-way ANOVA (Tukey’s test), n = 5 independent experiments, * *p* ≤ 0.05; *** *p* ≤ 0.001. ns, not significant.

As expected, eNK do not express HER2 or prostate-specific membrane antigen (PSMA) (**Figure 1A**). When eNK were incubated with BT-20 and SKBR3 cells, they gained HER2 expression (**Figure 1A**), although the levels were 10 times lower that those found in target cells (**Figures S1**, **1B**). eNK also gained PSMA when incubated with LNCaP cells that largely express this Ag (**Figures 1A, C**). Again, the expression of PSMA was 10 times lower for eNK than for LNCaP (**Figure 1C**). Optimal trogocytosis on all cell lines was observed at 3:1 E:T ratio (**Figures 1D**, **S3**). Of note, eNK encountering BT-20 cells acquire less HER2 expression than those encountering SKBR3 (**Figure 1A**, **S3**), although the tumor cell sensitivity to NK cytotoxicity was similar (**Figure S2**).

After confirming that NK cells can capture tumor antigens from solid cancer cells, we wanted to elucidate whether this gaining of expression was caused by trogocytosis. For this objective, as trogocytosis is a very rapid process, we restricted our co-cultures down to 2 h of incubation (38, 39). There is a lack of specific inhibitors for trogocytosis, but it has been described its interference by disruption of actin polymerization, inhibition of kinases (such as Src-kinase and Syk-kinase) and low temperature (4°C) (40, 41). Before performing the co-culture with SKBR3 tumor cells, we pre-treated eNK cells with cytochalasin-D (CytD) for inhibition of actin recruitment, or incubated the cells in the presence of PP2 for inhibition of Src-tyrosine kinase, which resulted in a decrease of HER2 acquisition by NK cells (**Figure S4A**). Moreover, we carried out these co-cultures at 4°C which completely reduced tumor marker acquisition (**Figure S4B**). We complemented these observations by using MDA-MB-468 as breast cancer donor tumor cells, which are negative for HER2 in comparison to SKBR3 (**Figure S4C**). Consistent with the idea that HER2 is acquired from donor cells via trogocytosis, NK cells co-cultured with MDA-MB-468 cells at different ratios did not increase HER2 expression after incubation (**Figure S4D**). However, this result would not be due to the absence of trogocytosis from this cell line, as we confirmed by membrane dye transfer experiments, in which NK cells were co-cultured with SKBR3 or MDA-MB-468 breast tumor cells previously labeled with the lipid intercalant dye DiD (**Figure S4E**). Even only after 2 h of co-culture, NK cells became strongly positive for this dye, and pre-treatments with CytD and PP2 along with 4°C incubation blocked the acquisition of donor membrane lipids from NK cells (**Figure S4E**). Considering that transfer of proteins via trogocytosis goes together with transfer of membrane lipids, altogether these results suggest that HER2 and other solid tumor markers are acquired by NK cells via trogocytosis.

**Figure 2:**
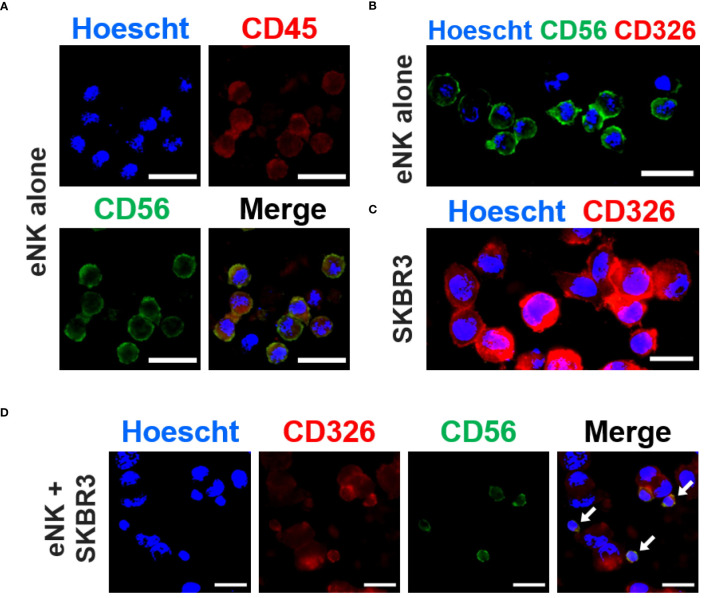
eNK cells capture solid tumor cell antigens *in vitro*. *In vitro* expanded NK cells (eNK) were incubated overnight alone or with SKBR3 solid tumor cells and, after antibody staining, the expression of several markers was analyzed by fluorescence microscopy. **(A)** eNK cells were stained with PE-conjugated anti-CD45 (red) and AlexaFluor®488-conjugated anti-CD56 (green) antibodies. The nucleus was stained with Hoescht (blue). **(B)** eNK cells were stained with PE-conjugated anti-CD326 (red) and AlexaFluor®488-conjugated anti-CD56 (green) antibodies. The nucleus was stained with Hoescht (blue). **(C)** SKBR3 cells were stained with PE-conjugated anti-CD236 (EpCAM, red) antibodies and Hoescht. **(D)** eNK cells co-cultured with SKBR3 cells at E:T ratio of 1:1 were stained for CD326 and CD56 as previously described. The representative micrograph panel show acquisition of CD326 expression by eNKs (yellow) via trogocytosis (white arrows). Scale bars (white): 20 µm.

To further confirm that eNK cells captured tumor cell-expressed receptors by trogocytosis, we analyzed the interaction between solid tumor cells (donor cells) and eNK cells during co-culture by the approach of fluorescence microscopy. As expected, we observed that eNK cells were positive for CD56 and CD45, and negative for EpCAM (**Figures 2A, B**), while solid tumor cells SKBR3 (donor cells) expressed EpCAM (CD326, **Figure 2C**). eNK co-cultured with SKBR3 cells interacted with them by forming immune synapses and performing cytotoxicity. After trogocytosis *in vitro*, eNK acquired surface expression of EpCAM. (**Figure 2D**), which was absent in NK cells incubated alone. This surface expression was still evident after several washing steps (**Figure 2D**).

**Figure 3:**
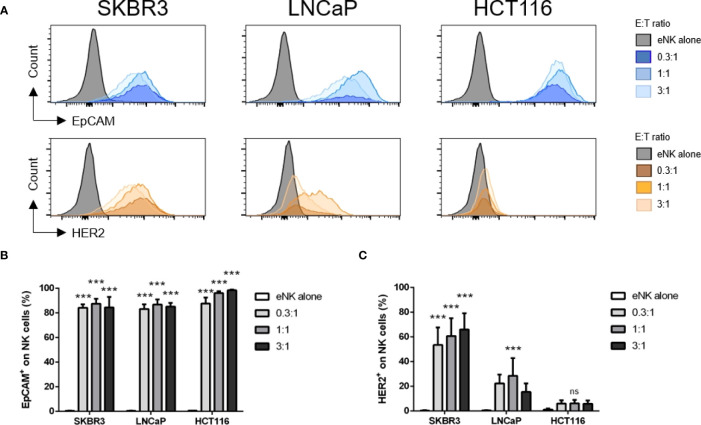
Different Ags are differently trogocytosed from different cell lines. *In vitro* expanded NK cells (eNK) were incubated alone or at different effector:target (E:T) ratios with solid tumor cells, and after exclusion of doublets and dead cells, the surface expression of HER2 and EpCAM was analyzed on live CD56+CD335+ NK cell population. **(A)** Representative histograms depict EpCAM (CD326) and HER2 expression on eNK cells. **(B, C)** Bars represent mean ± SEM of n = 4 independent experiments. Statistical significance between untreated cells and different E:T ratios were determined by two-way ANOVA (Tukey’s test); *** *p* ≤ 0.001. ns, not significant.

We next investigated if different Ags were differently uploaded from the same targets. eNK efficiently gained EpCAM, but much less HER2, from HCT116 or LNCaP cells (**Figure 3**), whereas these cells express substantial amounts of both Ags (**Figure S1**). But this difference does not depend on HER2, because both HER2 and EpCAM are efficiently uploaded from SKBR3 cells (**Figure 3**). In summary, the efficiency of trogocytosis is variable regarding to the target cell, the efficiency of killing and the type of Ag.

### Identification of trogocytosis *ex vivo*

We used a cohort of patients operated for a breast tumor (8 invasive breast cancers, one ductal *in situ* carcinoma, one atypical ductal hyperplasia) to analyze several phenotypic markers on peripheral NK cells. We collected and analyzed blood samples at diagnosis and compared them to NK cells of a cohort of 5 healthy donors (HD) by multiparametric flow cytometry (**Figure S5**). Cancer patients expressed different values of estrogen and progesterone receptors and few of them overexpressed HER2. For future analysis in this manuscript, we could independently analyze patients with high expression of estrogen and/or progesterone receptors (patients 2, 3, 4, 5, 8, 10; receptor-positive or Receptor+ or R+) and those with low or unknown expression (patients 1, 6, 7, 9; receptor-negative or Receptor- or R-). Of note, estrogen and progesterone receptors are nuclear proteins and hence, NK cells should not perform trogocytosis on them.

**Figure 4:**
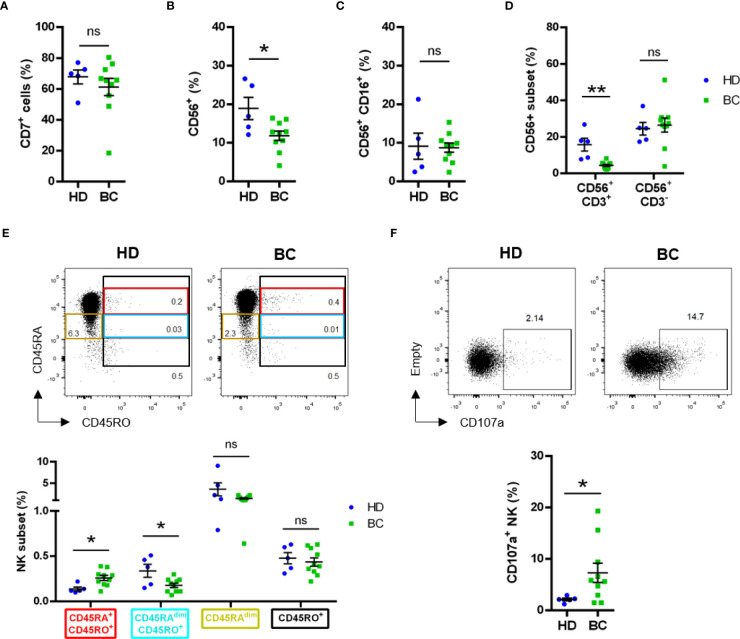
Phenotype analysis of peripheral NK cells analyzed by manual gating. Blood samples from healthy donors (HD) and breast tumor (BC) patients were analyzed by FACS for expression of lymphoid and NK cell subset markers. Graphs represent compiled data of **(A)** frequency of live CD7+ cells; **(B)** frequency of live total CD56+ cells and **(C)** frequency of live total CD56+CD16+ cells. **(D)** Frequency of live CD56+CD3- or CD56+CD3+ between HD and BC patients. **(E)** Frequency of live CD3-CD4-CD7+CD56+ NK subsets based on CD45RA and CD45RO expression between HD and BC patients. **(F)** Expression of CD107a on live CD3-CD4-CD56+CD7+ NK cells. Graphs represent mean ± SEM; statistical significance between HD (n = 5) and BC (n = 10) was determined by Student t-Test **(A–C, F)** or two-way ANOVA **(D, E)**; * *p* ≤ 0.05; ** *p* ≤ 0.01. ns, not significant.

The percentage of CD7^+^ cells, which mainly include T and NK cells tended to decrease in patients; and in fact, the percentage of CD56^+^ cells decreased (**Figures 4A, B**). However, the CD56^+^/CD16^+^ cells, which represents the mature NK cells, remained stable (**Figure 4C**). The CD56^+^ cell subset that decreased were CD3^+^ and should represent NK T cells (**Figure 4D**). In contrast, the CD56^+^/CD3^-^ population, which represents NK cells, remained stable.

In hematological cancers, the main antitumor NK cell population is recognized by the expression of CD45RO (CD45RO cells), generally together with CD45RA (CD45RARO cells (23–26);. We observed a small, but significant, increase of this CD45RARO population in peripheral blood NK cells of patients (**Figure 4E**). This was associated with a decrease in cells expressing low CD45RA levels (CD45RA^dim^) and CD45RO (CD45RA^dim^RO cells). On the other side, the CD45RO^+^ NK subset frequency was found similar between BC patients and HD (**Figure 4E**).

We next evaluated degranulation, i.e. CD107a^+^ cells, in the lymphoid, i.e. CD7^+^, compartment and observed an increase in patients (**Figure S6A**). Exclusion of CD14^+^/CD19^+^ cells did not change our observation (**Figure S6B**). The differences were not statistically different when we focused on receptor-negative patients (**Figure S6C**) and hence, the increase mainly relied in receptor-positive patients (**Figures S6D, E**). Analysis of *bona fide* CD7^+^CD56^+^CD3^-^ NK cells (42) confirmed the higher degranulation of NK cells in BC patients (**Figure 4F**).

**Figure 5:**
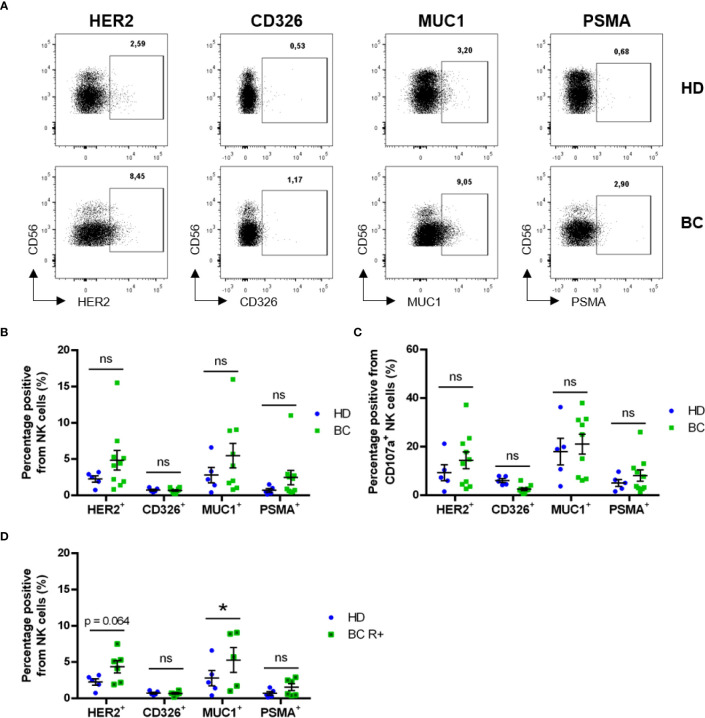
Circulating NK cells exhibit trogocytosis of solid tumor-expressed markers. Blood samples from healthy donors (HD) and breast cancer (BC) patients were analyzed by FACS for expression of tumor cell markers on CD7+CD56+ NK cell subsets. **(A)** Representative dot plots showing expression of tumor markers in circulating NK cells. **(B)** Frequency of NK cells presenting surface expression of HER2, CD326 (EpCAM), MUC1 or PSMA between HD and BC patients. **(C)** Frequency of trogocytosed markers on CD107+ NK cells. **(D)** Frequency of NK cells presenting surface expression of tumor markers in Receptor-positive patients (BC R+) versus HD. Graphs represent mean ± SEM; statistical significance between HD (n = 5) and BC (n = 10), BC-R+ (n = 6) and BC-R- (n = 4) was determined by two-way ANOVA **(B–D)**; * *p* ≤ 0.05. ns, not significant.

We analyzed trogocytosis of 4 endothelial/tumor markers, which are not expressed by NK cells. These were: HER2 (human epidermal growth factor receptor 2, which is overexpressed in certain patients), CD326 (EpCAM), Mucin 1 cell surface associated (MUC1) and PSMA. The membrane localization of all these proteins makes them candidates to be trogocytosed and exposed in NK cell plasma membrane. Patients showed higher trogocytosis of all markers except EpCAM (**Figure 5A**), suggesting that this Ag is not well uptaken by NK cells *in vivo*. However, differences were not statistically significant in the total NK cell population (**Figure 5B**), nor in the cells that had degranulated (**Figure 5C**). Notably, when we focused the analysis on receptor-positive patients, the frequency of total CD7+CD56+ NK cells exhibiting the tumor markers MUC1 and HER2 appeared to be increased (**Figure 5D**). The percentage of cells that have performed trogocytosis on the epithelial markers was significantly higher in CD107a^+^ NK cells than in the bulk of NK cells, principally in patient’s samples (**Figure S6F**). We also observed this increase in CD45RARO cells regarding the bulk of NK cells (**Figure S6G**). However, we did not observe significant differences between HD NK CD45RARO+ and BC NK CD45RARO+ populations, nor between CD45RARO+CD107a^+^ NK cells from both groups (**Figure S6H**). This indicates that CD45RARO and CD107a^+^ cells are the NK cell populations that are interacting with target cells and recovering antigens by trogocytosis. Although there were not significant differences between receptor-negative BC patients and HDs, some of the tumor markers analyzed were found upregulated on CD7+CD56+ NK cells from receptor-positive BC patients when compared with HD (**Figure 5D**). The fact that HDs show a similar pattern suggests that NK cells can interact and probably kill endothelial cells, which could be stress or damaged cells.

### Differential trogocytosed-receptor expression pattern in NK cell subsets between BC patients and healthy donors by viSNE

In order to further characterize the expression of these tumor markers present on NK cell surface due to trogocytosis, we used the high-dimensional reduction algorithm viSNE (for visualization of t-Distributed Stochastic Neighbor Embedding or t-SNE) (35, 43). By viSNE, we generated unsupervised 2D t-SNE maps showing the expression of 12 surface markers on NK cells from BC patients and healthy donors (**Figure S7**). The resulting t-SNE maps exhibit several spatial regions with differences in marker expression, suggesting that NK cell subsets with different abundance and expression of those markers were present.

**Figure 6:**
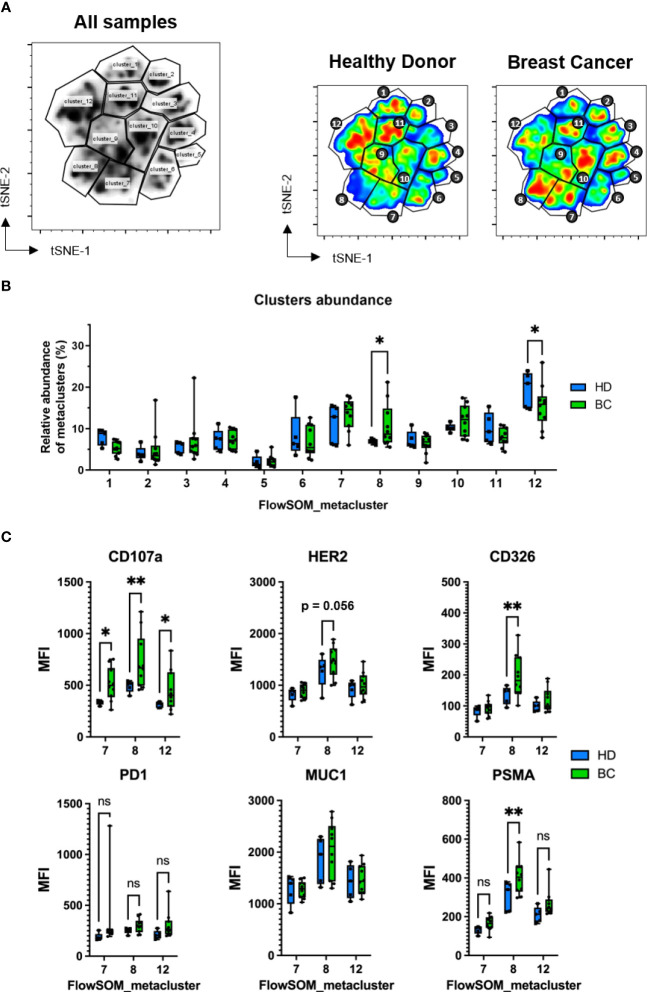
Identification of NK cell clusters with acquired tumor markers by FlowSOM. FACS samples from 5 healthy donors and 10 BC patients were concatenated and randomly subsampled into 100.000 total events which were analyzed by t-SNE and displayed using viSNE. **(A)** FlowSOM-identified twelve metaclusters visualized in the concatenated file. To the right, metacluster-derived gating applied to concatenated HD and BC patient samples. Differences in color on the viSNE map correspond to cell abundancy density. **(B)** Comparison of relative frequency of each FlowSOM metacluster between HD and BC patients. **(C)** Median fluorescence intensity (MFI) of CD107a, PD1 and trogocytosed tumor markers expressed on CD7+CD56+ NK cells present on selected metaclusters (7, 8 and 12). Graph represent box and whiskers (Min to Max), and statistical significance between HD (n = 5), BC (n = 10) was determined by two-way ANOVA; * *p* ≤ 0.05; ** *p* ≤ 0.01. ns, not significant.

To evaluate the differences in NK cell subset distribution between BC patients and healthy donors, we applied the FlowSOM method which uses Self-Organizing Maps (SOMs) to cluster together cell events based on clustering channels (markers) and assign them to metaclusters, grouping them into distinct populations by an unsupervised approach (36). By this method we identified twelve metaclusters, of which two, i.e. 8 and 12, were found to be differentially abundant between BC patients and healthy donors (**Figures 6A, B**, **Table S1**). Both mainly consisted in NK cells with phenotype CD56^+^CD16^high^CD45RA^+^ CD107a^+^ and tumor markers expression (**Table S1**), Metacluster-8 was found enriched in BC patients in comparison with healthy controls and contained CD45RO^+^ cells (10.7% versus 6.9% of control, p<0.05) (**Figure 6B**). This difference was even more evident when we compared receptor-positive patients with healthy controls (12.4% versus 6.9% of control, p<0.01) (**Figure S8A**, **Table S1**). On the other side, metacluster-12 was found to be decreased in BC patients compared to healthy donors (15.5% versus 19.5% of control, p<0.05). This difference was higher when controls were compared with receptor-negative patients (13.2% versus 19.5% of control). In contrast to metacluster-8, this metacluster-12 mostly comprised CD45RO^-^ NK cells (**Table S1**).

When we analyzed the degranulation on NK cells, we found that CD107a expression levels on Metacluster-7, Metacluster-8 and Metacluster-12 were significantly higher in BC patients compared to controls (**Figure 6C**). CD107a+ was also found increased in NK cells from Metacluster-10 when comparing receptor-positive BC patients and healthy donors (**Figure S8B**). Thus, considering that NK cells having a CD45RARO/CD107a+ phenotype previously exhibited higher degree of presumed trogocytosed proteins, we analyzed the expression levels of these solid tumor markers on these metaclusters. Interestingly, among all FlowSOM-identified metaclusters, we found that NK cells contained in Metacluster-8 showed the higher levels of tumor markers and, compared to healthy controls, BC patients exhibited significantly higher levels for the tumor markers CD326 and PSMA (**Figure 6C**). Moreover, HER2 was found particularly increased on NK cells from Metacluster-8 of receptor-positive BC patients in comparison to healthy donors (**Figure S8B**). Lastly, the expression of the immune checkpoint receptor programmed cell death-1 protein (PD1) was also analyzed on these NK cells, and when we compare its expression between healthy control and BC samples (total or receptor-positive patients), there was not significative difference between both groups (**Figures 6C**, **S8B**). Altogether, these results suggest that *bona-fide* NK cells with an activated phenotype (CD107+ CD45RARO) are found increased on BC patients and, additionally, these NK cells exhibit high degree of potentially trogocytosed tumor markers on their surface.

**Figure 7:**
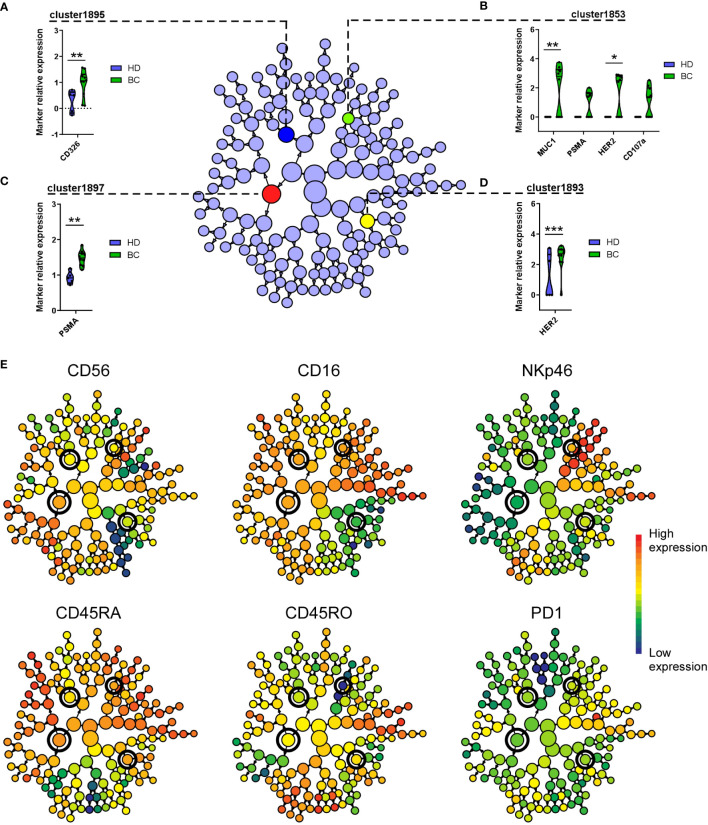
CITRUS identifies NK cell clusters with differential expression of tumor markers on BC patients. Cluster identification, characterization, and regression (CITRUS) algorithm identifies cell subsets (clusters) with significantly differential marker expression between BC patients and HD. Graphs displayed as CITRUS maps show the normalized expression (arcsinh) of CD107a and trogocytosed tumor markers on Metacluster-8 NK cells contained in **(A)** cluster-1895, **(B)** cluster-1853, **(C)** cluster-1897 and **(D)** cluster-1893, displayed as violin plots. **(E)** CITRUS maps overlaid with marker-intensities show relative expression of mentioned phenotype markers. Highlighted nodes in CITRUS maps correspond to cell subsets with differential tumor marker expression obtained using a significance analysis of microarray (SAM) correlative association model (Benjamini-Hochberg, adjusted *p-*value < 0.01). * *p* < 0.05, ** *p* < 0.01, *** *p* < 0.001.

To complement these observations with FlowSOM analyses, we further examinate the cell activation and trogocytosis profile of BC patients and controls, focusing on NK cells contained in Metacluster-8 and using CITRUS (37). This algorithm allows the identification of stratifying sub-populations in multidimensional flow cytometry datasets, and can be used to distinguish single-cell signatures that might be associated with clinical outcomes (44). Among NK cells in Metacluster-8, CITRUS identified a total of 153 clusters, of which four were found to be statistically associated to the BC patient’s group, in accord to their CD107a and tumor marker expression (**Figures 7A-D**). By associating this CITRUS map with maps showing surface phenotype markers intensities (**Figure 7E**), we could identify these clusters specifically as subsets of CD56+ NK cells expressing high levels of CD16, CD45RA, NKp46 and intermediate levels of CD45RO, with high expression of CD326, PSMA, HER2 and MUC1 (**Figure 7**).

## Discussion

Individual humans have their own NK cell repertoire, which changes during development and is different in diverse tissues. In addition, multifactorial environmental events affect NK cells and generate new subsets (45) and make challenging to identify NK populations associated to a specific disease and shared by multiple patients. Conventional NK cells, which should correlate with the population that we have studied, have a short half-life (5, 46). Hence, the amount of NK cells that has infiltrated the tumor and interacted with targets, then performed trogocytosis and came back to the blood stream should be scarce. In this current work, we have found a very low number of NK cells carrying tumor Ags, showing that their numbers are very low or, alternatively, to find and identify them is extremely difficult. Moreover, we observed that NK cells from HD also carry some Ags typical of endothelial cells that we have used as trogocytosis markers. Probably, these NK cells have interacted with “stressed” endothelial cells. In case of breast cancer patients, the amount of “stressed” endothelial cells should be larger, because tumor cells are supposed to be stressed and recognized by NK cells, increasing the portion of NK cells that carry endothelial markers. However, most of the NK cells that have interacted with tumor cells should be in the tumor microenvironment as shown previously (47). Here we show that effectively their frequency in periphery is very low, and to find differences between HD and patients we needed to unveil new populations (**Figures 6**, **7**). Of note, NK cells eliminate senescent cells, which are considered “stressed”, favoring the clearing of this population and keeping an adequate cell population (48). In support of this hypothesis, NK cells carrying endothelial markers have predominantly degranulated, i.e. they are CD107^+^. This observation is consistent with previous reports describing higher degranulation and expression of activation markers on NK cells that acquired tumor cell markers by trogocytosis, proposing the surface expression of trogocytosed tumor markers as indicative of contact between these NK cells and marker-positive cancer cells (47, 49).

Our observations from *in vitro* experiments strongly imply that transference of HER2, EpCAM and other solid tumor cell markers on NK cells occurs via trogocytosis, proposing this process as the mechanism of capture of epithelial and tumor markers by NK cells under physiological and pathological conditions, as reported in tumor biopsies from BC patients (47). However, it is not possible to definitively conclude that HER2 and the rest of tumor markers found on circulating NK cells from BC patients would have been transferred via trogocytosis. These membrane proteins are normally absent and not expressed by NK cells, and our unsupervised multiparametric analyses found specific subsets of NK cells with high surface levels of these epithelial and solid tumor markers present in blood samples of BC patients; therefore, this correlation should not be completely ruled out, this point remaining pending of further elucidation.

In our study we focused on *bona fide* NK cells (CD7^+^ CD56^+^ CD3^-^) from patient’s peripheral blood. It has been reported that tumor-infiltrating monocytes and NK cells that can be found in breast tumors could show high HER2-trogocytosis, mainly when patients are treated with mAbs systemic treatment, which facilitate NK cell recruitment and activation (47). These observations make us suggest that the amount of Ag capture by trogocytosis on tumor-infiltrating NK cells could be higher and more measurable than the surface levels detected on circulating NKs. However, analysis of infiltrated, tissue-specific human NK populations could prove more challenging and complex (45). For this reason, we did not investigate tissue-specific NK cells that would require biopsies. Hence, we have not probably identified all disease-associated NK populations, but only those that could exit the tumor environment. In addition, we have used a defined panel of NK-associated markers, whereas NK cells can express hundreds of them (50). In summary, other anti-tumor NK populations probably exist and perform degranulation and trogocytosis.

The identification of subsets of circulating NK cells, consisting of activated cells with evident degranulation carrying epithelial and tumor cell markers, raises the issue of the relevance that holds this tumor-trogocytosed marker harboring on NKs cellular and immune functions. Several reports in mice observed that MHC-I acquisition from target cells decreases acceptor NK cell immune recognition and cytotoxic functions (51–54). Correspondingly, it was reported that human NK cells that capture HLA-G (a non-classical MHC-I immunosuppressive molecule) from melanoma solid tumor cells not only massively reduce their proliferation and their cytotoxicity, but also are able to suppress the cytotoxic function of other bystander NK cells expressing the HLA-G ligand and inhibitory receptor ILT2 (14). Similarly, trogocytosis of CD9 molecules from ovarian carcinoma cells to NK cells renders them less cytotoxic and poorer producers of anti-tumor cytokines, consistent with the identification of a CD9-positive NK cell subset in tubo-ovarian carcinoma samples which presence correlates with tumor progression (55). In line with this, a recent report showed that murine NK cells can acquire by trogocytosis the immune checkpoint inhibitor programmed cell death protein 1 (PD1) from leukemia cells both *in vitro* and *in vivo*, suppressing anti-tumor NK cell immunity (20).

However, this might not be the case for tumor receptors like HER2, because similarly to our present results, it has been observed that NK cells co-cultured with trastuzumab-opsonized HER2+ breast cancer cells can acquire HER2 receptor via trogocytosis and exhibit higher expression of CD107a than non-HER2-trogocytosed NK cells (47). In addition, NK cells are capable to obtain the tyrosine kinase receptor TYRO3 from leukemia cells *in vitro* and *in vivo*, displaying higher levels of activation markers, enhanced cytotoxicity and interferon-γ secretion (49), thus providing opposing evidence that tumor receptor trogocytosis on NK cells could also translate into gain of anti-tumor activity and effector function (56). This study was focused on the identification of NK cell subsets harboring trogocytosed markers from breast cancer cells, and even if experiments to elucidate the functional outcome of the acquisition of these markers by trogocytosis are pending to perform, we observed high levels of CD107a and low levels of PD1 expression on these NK cells from BC patients, which considering the mentioned literature is consisting with high anti-tumor function of these NK cells.

We have found also that degranulation and trogocytosis is higher in patients that expressed estrogen and/or progesterone receptors. This could be related to the main historical view that triple-negative breast cancer (TNBC) is a cold tumor (57). Hence, it is expected that NK cell infiltration and interaction with tumor target cells is poor in these patients. This should explain the lower level of NK cells that have degranulated and performed trogocytosis in peripheral blood from these patients in our study. However, a larger cohort of patients including both estrogen/progesterone receptor-positive and -negative would be necessary to conclude with greater certain this point.

We expanded our observations by performing dimensionality reduction analysis with viSNE, and we complemented it by using FlowSOM and CITRUS algorithms to further determine NK cell subsets carrying tumor cell antigens. Our unsupervised analyses identified clusters over-represented in BC patients containing tumor markers-expressing NK cells, i.e. metacluster 8. This cluster consisted mostly of CD45RARO+ cells, with high level of degranulation that acquired surface expression of tumor markers, most probably by trogocytosis. These cells also exhibited relative expression levels of PD1, although we found no significative difference for this marker between BC and HD group (p = 0.61). Complementing this information, we have observed a very low PD1 levels (from 1% to 5% positive tumor cells) in our cell lines. This could suggest that PD1 is probably from NK cell origin in our settings, in accord with previous reports (58). Conversely, we cannot definitively rule out the likeliness that PD1 expression detected on NK cells derives from cancer cells, considering that PD1 expression on tumors derived from BC patients included in this study was not determined, and it has been already described that in different experimental settings NK cells can gain also PD1 by trogocytosis (20). Therefore, one possibility is that PD1 expression could increase on NK cells once they have acquired cancer cell markers. This could be due to NK cell activation after recognition of target cells, which have been reported on both circulating and tumor-infiltrating NK cells from several types of solid tumors (59–62). We favor this hypothesis, but we cannot exclude that PD1+ NK cells are more prone to acquire cancer markers. The physiological relevance could be important, because PD1+ NK cells with cancer antigens may participate in maintaining an immunosuppressive state and affect some immunotherapy treatments. Thus, trogocytosis of these types of receptors can show strong immunomodulatory capacities on NK cell immune function and capabilities.

Finally, our *in vitro* approach shows that different Ags are differently trogocytosed from different target cells and this is independent of the sensitivity to NK cells. NK cells extract for example HER2 and EpCAM from SK-BR3 or LNCaP cells; but almost exclusively EpCAM from HCT116 or BT20 cells. Meanwhile, the sensitivity of all these cell lines to NK cells was comparable. Hence, which molecules are trogocytosed depend on the donor cell and the nature of the molecule. The understanding of the precise mechanism of trogocytosis will be essential to unveil the reasons of the disparity between Ags and donor cells.

## Data availability statement

The original contributions presented in the study are included in the article/**Supplementary Material**. Further inquiries can be directed to the corresponding author.

## Ethics statement

The studies involving human participants were reviewed and approved by ICM-BDD 2017/37 (ID-RCB: 2017-A01940-53) clinical program approved by the “Comités de Protection des Personnes Sud-Ouest et Outre-Mer III”. The patients/participants provided their written informed consent to participate in this study.

## Author contributions

WJ provided essential material and follow patient’s status. GG, M-LD and MC performed the experiments. MC-M and MV performed study design and wrote the article. All authors contributed to the article and approved the submitted version.
