# Supplementary material for: NK cells in peripheral blood carry trogocytosed tumor antigens from solid cancer cells
Source: Front Immunol. 2023 Aug 1;14:1199594. doi: 10.3389/fimmu.2023.1199594 (PMC10427869; doi:10.3389/fimmu.2023.1199594)
Supplement: Supplementary file 1 [file DataSheet_1.pdf]

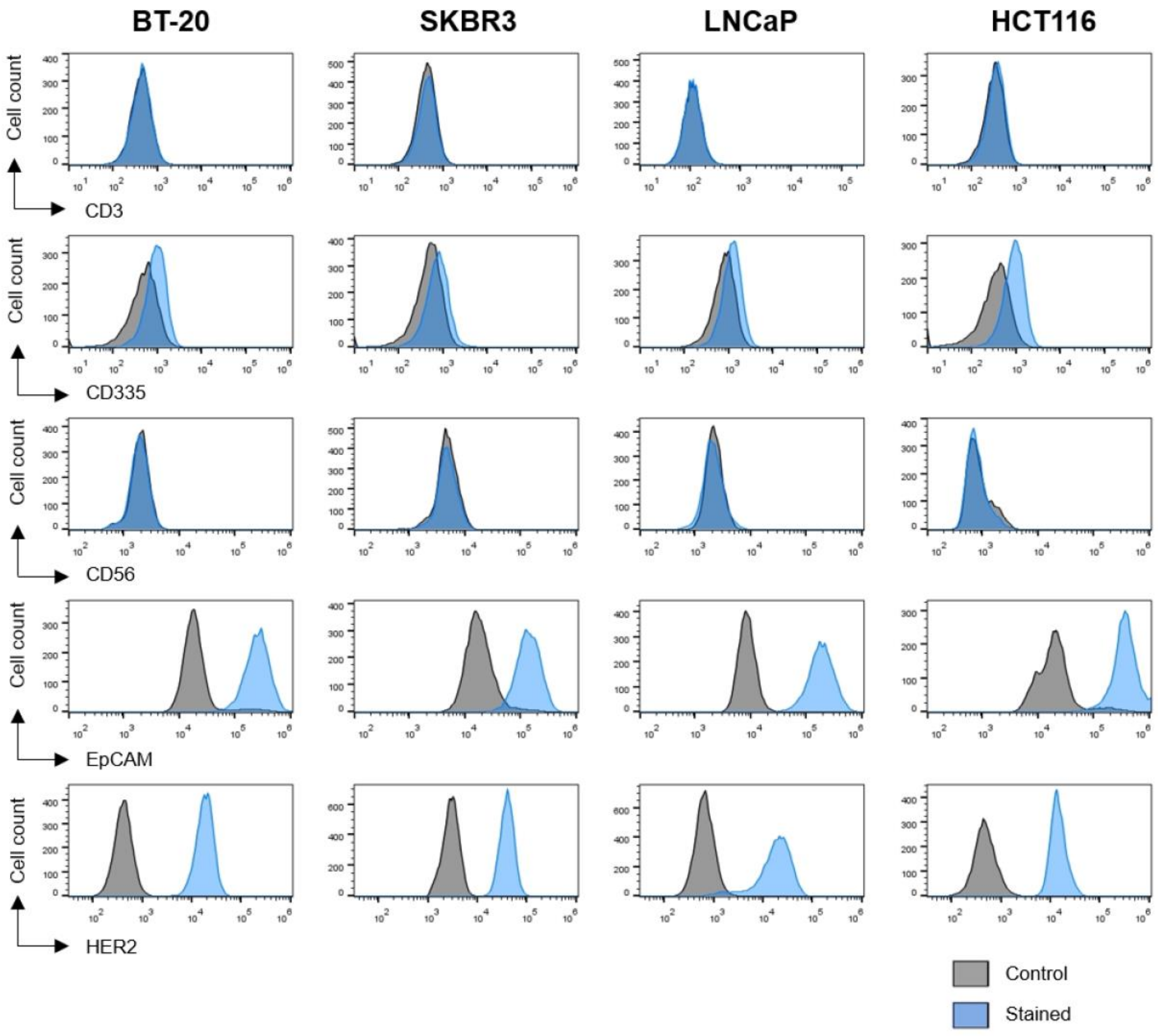

Figure S1. Expression of different antigens on solid tumor cell lines. The adherent tumor cell lines BT-20, SKBR3, LNCaP and HCT116 were stained with antibodies against CD3, CD56, CD335, HER2 and EpCAM before FACS analysis. Representative histograms depict fluorescence in cells stained with isotype control (dark grey) or each with fluorochrome-coupled antibody (light blue). Representative data of n = 2 independent experiments.

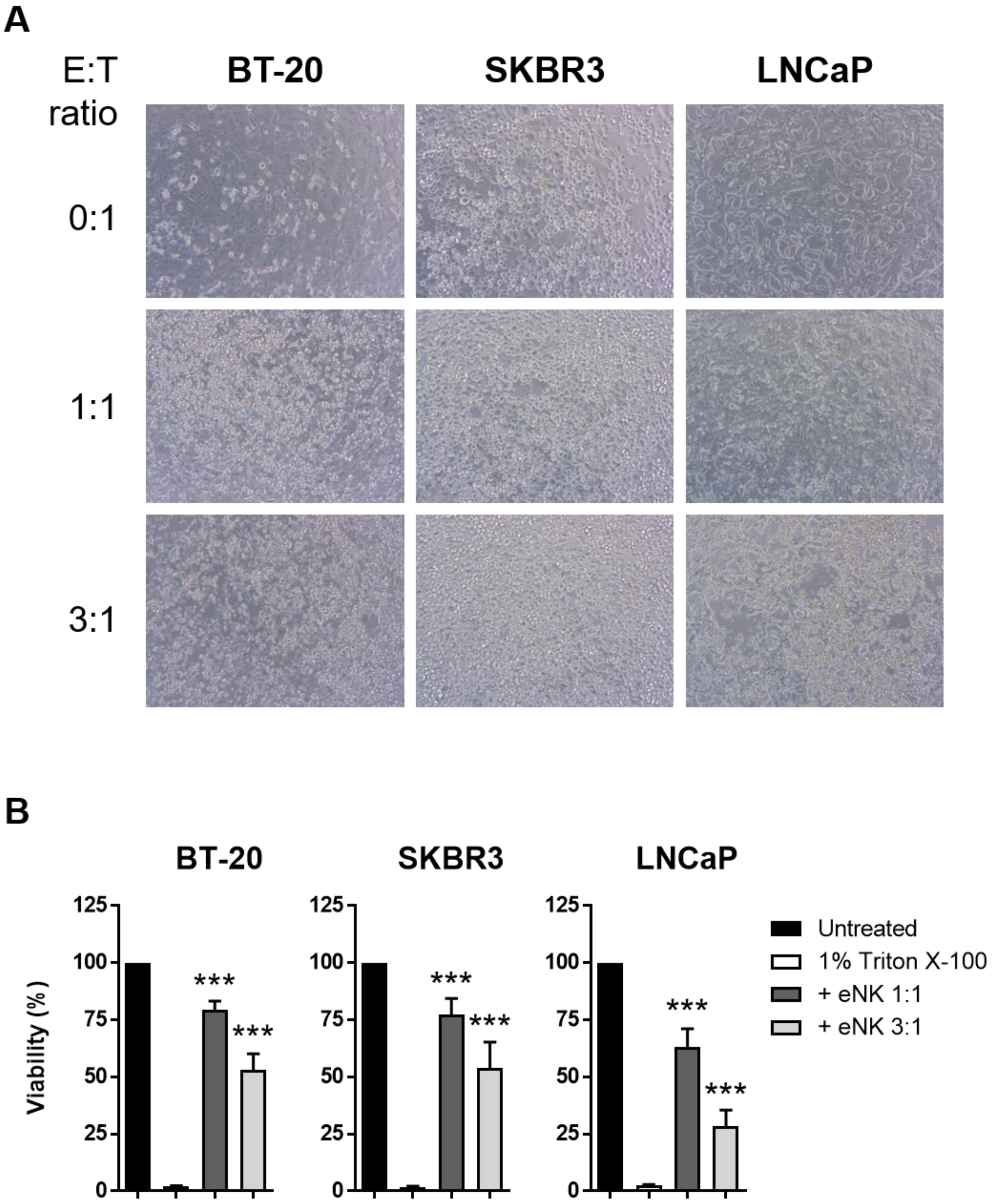

Figure S2. Solid tumor cell lines viability after NK cell treatment. Expanded NK cells were incubated with different tumor cell lines. **A)** Representative light fields of tumor cells co-cultured with eNK cells at different ratios (amplification of 20X). **B)** After overnight co-culture with eNK cells, tumor cell viability was determined by MTT assay. Representative graphs show mean±SEM of tumor cell viability (normalized to untreated cells). Statistical significance between untreated cells and different E:T ratios were determined by two-way ANOVA (Tukey's test); n = 5 independent experiments; *** $p \leq 0.001$.

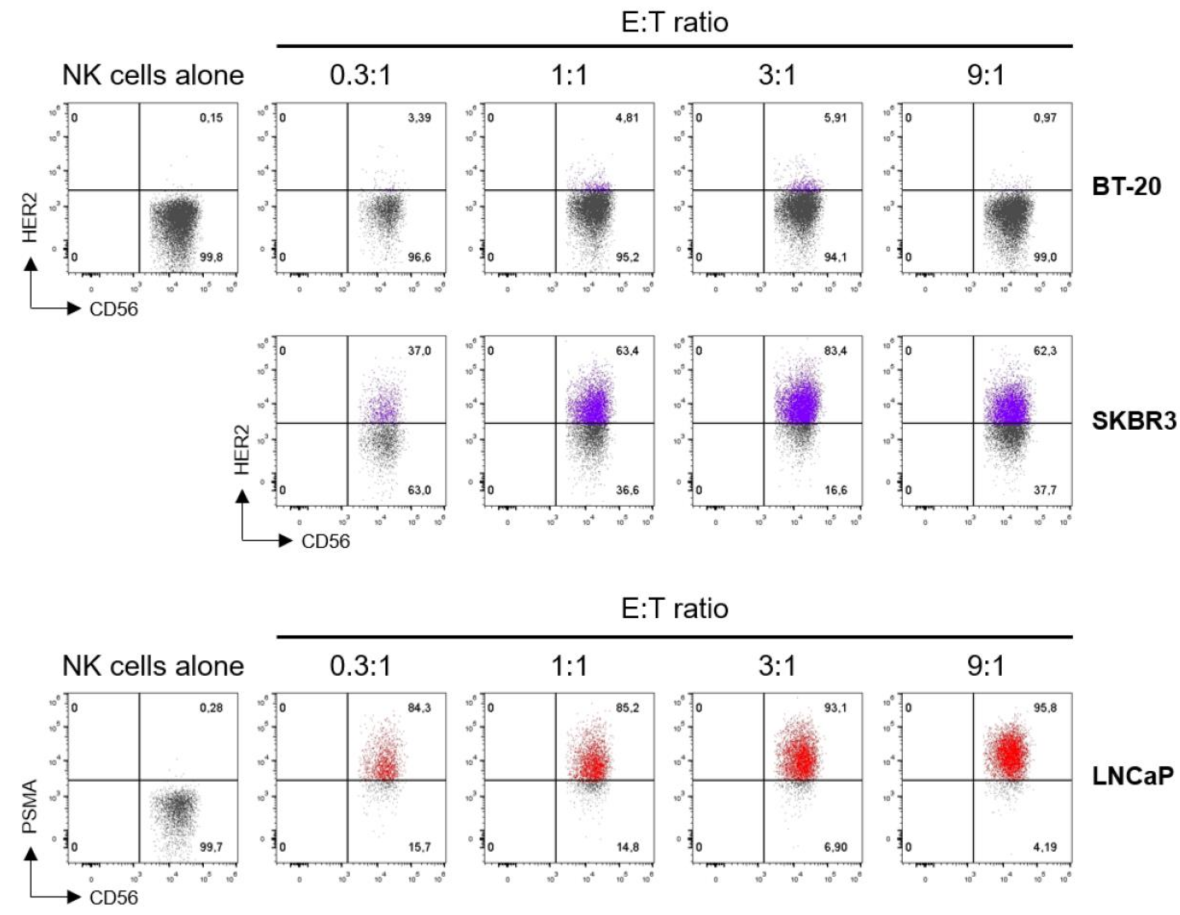

Figure S3. NK cells acquire solid tumor cell markers *in vitro* at different E:T ratios. In vitro expanded NK cells (eNK) were incubated alone or co-cultured at different effector:target (E:T) ratios with several solid tumor cells overnight. After antibody staining, the expression of tumor markers was analyzed on live CD3-CD56+CD335+ NK cell population. Representative dot plots show frequency of HER2 (for BT-20 and SKBR3) and PSMA (for LNCaP) in the surface of NK cells. n = 5 independent experiments.

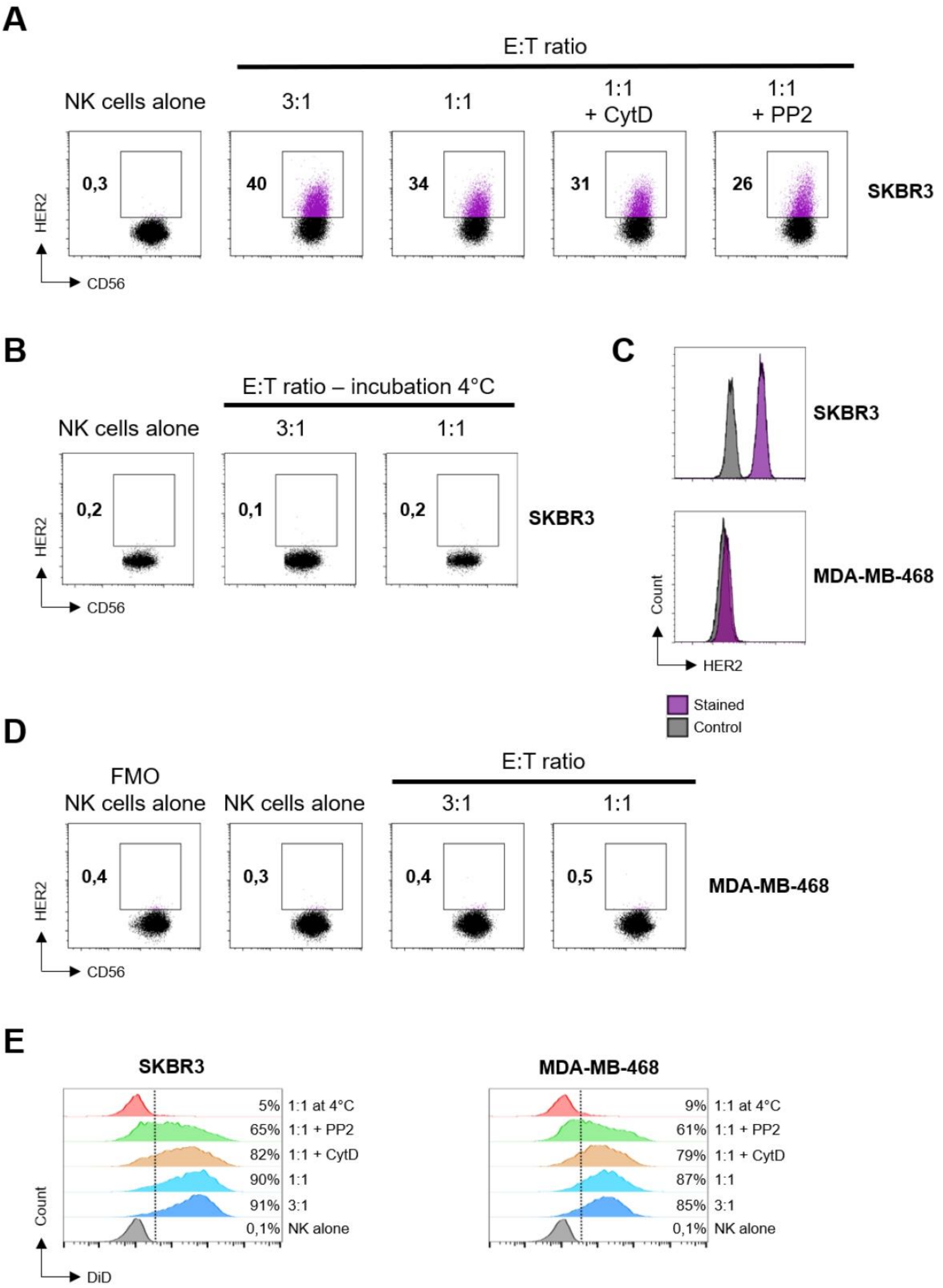

Figure S4. Transfer of HER2 and membrane lipids from tumor to NK cells occurs via trogocytosis. A) In vitro expanded NK cells (eNK) were incubated with the actin recruitment inhibitor cytochalasin D (CytD) or Src-kinase inhibitor PP2, and then cultured at different effector:target (E:T) ratios with SKBR3 cells for 2 h and, after antibody staining, the expression of HER2 (purple) was analyzed on CD3-CD56+ NK cell population. **B)** NK cells were co-cultured with SKBR3 cells at low temperature (4°C) for 2 h and HER2 expression analyzed by FACS. **C)** HER2 expression of SKBR3 and MDA-MB-468 breast cancer tumor cell lines. Gray,

isotype control; purple, PE-conjugated anti-HER2 antibodies. **D)** NK cells were co-cultured with MDA-MB-468 cells for 2 hours and HER2 expression was analyzed by FACS. **E)** SKBR3 and MDA-MB-468 cells were stained with lipid membrane dye DiD and co-cultured with NK cells. After 2 h, DiD acquisition by NK cells alone or under the mentioned conditions was measured by FACS. Representative dot plots and histograms from n = 2 independent experiments.

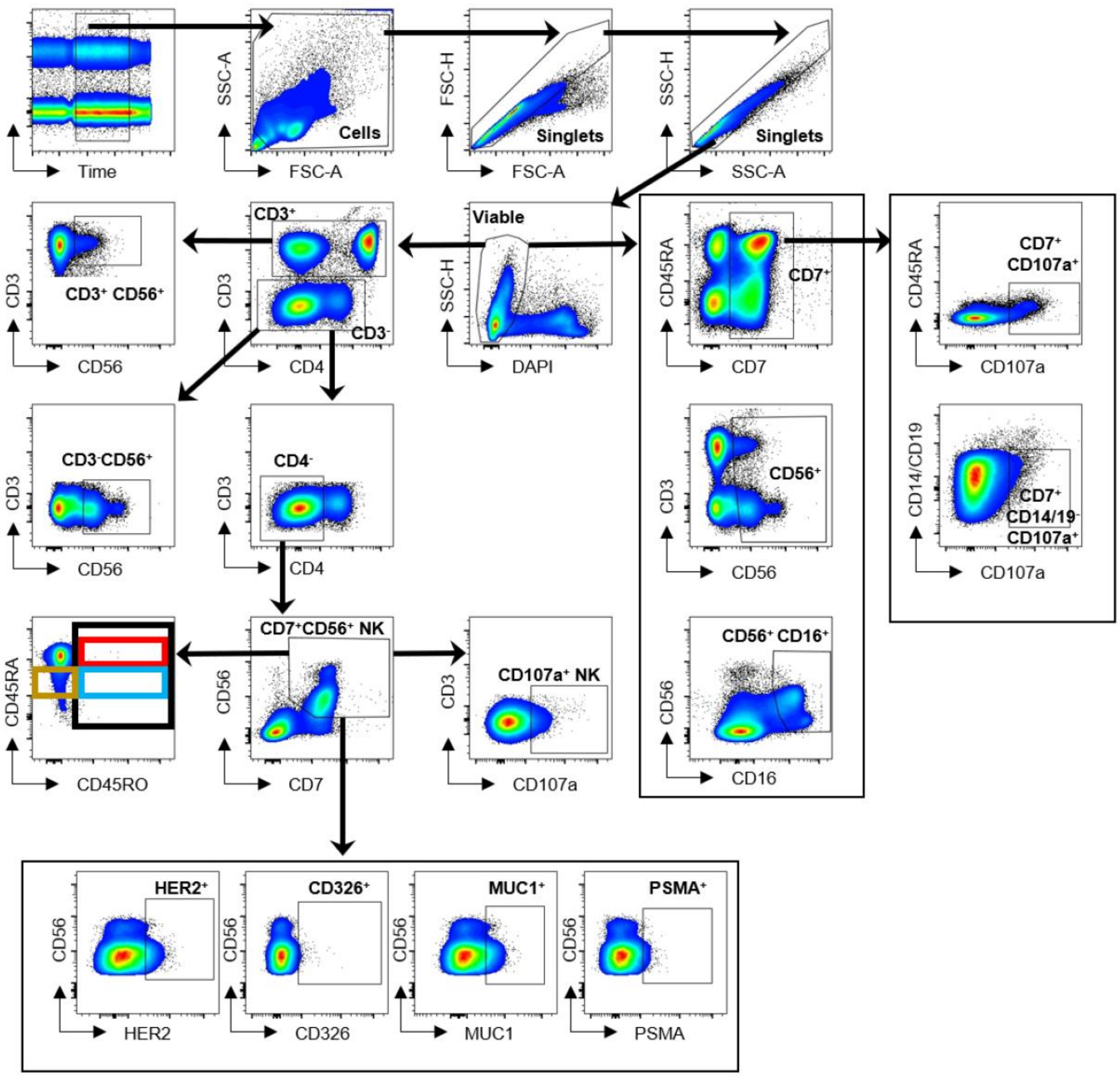

Figure S5. Manual gating strategy for conventional flow cytometry analysis. Representative FACS plots depicting manual gating for immune subsets in healthy donors or cancer patients. For the CD45RA vs CD45RO dot plot gates: total CD45RO+ (black gate); CD45RA+CD45RO+ (red gate); CD4RAdimCD45RO+ (blue gate); CD45RAdim (gold gate).

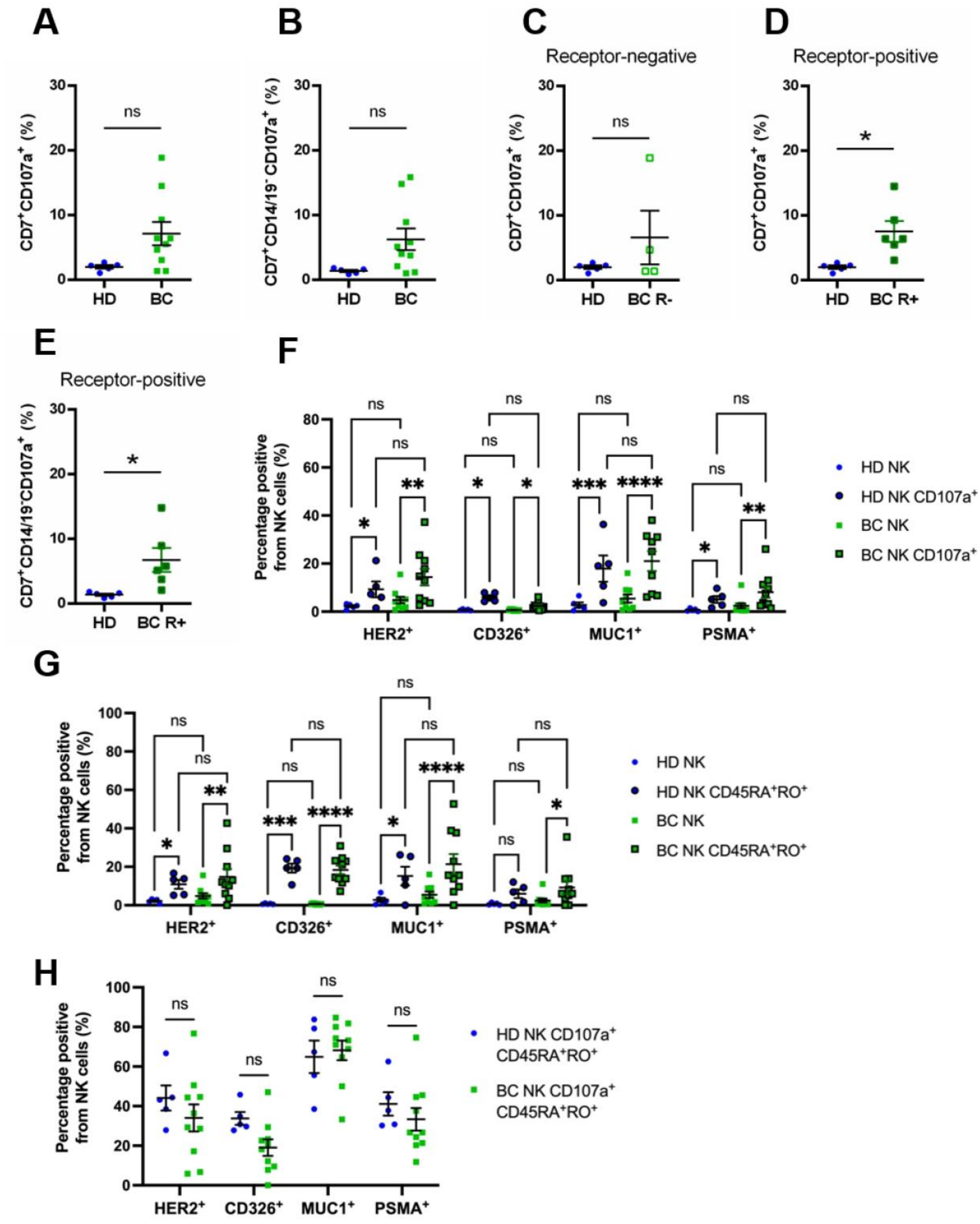

Figure S6. Differential phenotype of immune cell subsets based on patient's receptor expression. Blood samples from healthy donors (HD), total breast cancer (BC) patients and receptor-high (BC R+) or receptor-low (BC R-) BC patients were analyzed by FACS for expression of immune cell subset markers. Graphs represent compiled data of frequency of CD107a+ cells in the compartments CD7+ **(A)**, CD14-CD19-CD7+ **(B)**, CD7+ in the receptor-negative patients **(C)**, or in the receptor-positive patients from CD7+ **(D)** or CD14-CD19-CD7+ **(E)** cell subsets. **F)** Frequency of tumor marker expression on CD7+CD56+ NK versus CD7+CD56+CD107a+ NK cells in HD and BC patients. **G)** Frequency of trogocytosis on

CD56+ NK versus CD56+CD45RA+RO+ NK cells between HD and BC patients. **(H)** Frequency of trogocytosis on CD56+CD107a+CD45RA+RO+ NK cells between HD and BC patients. Graphs represent mean±SEM; statistical significance between HD (n = 5), BC (n = 10), BC-R+ (n = 6) and BC-R- (n = 4) was determined by Student t-Test (A-E) or two-way ANOVA (F-H); * $p \leq 0.05$; ** $p \leq 0.01$; *** $p \leq 0.001$.

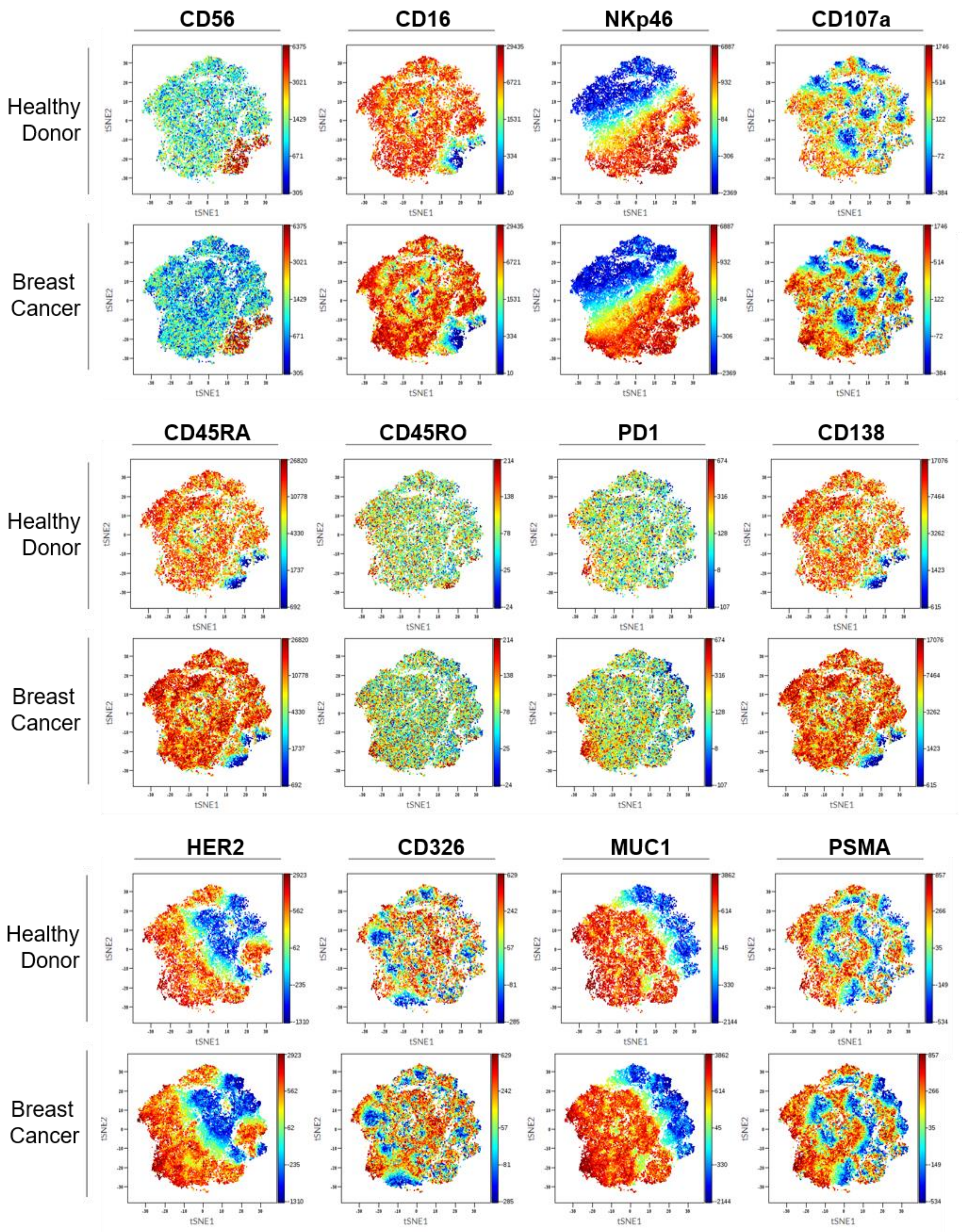

Figure S7. viSNE maps colored by channel intensities for the expression of immune and tumor markers between HD and BC patients. FACS samples from 10 BC patients were concatenated into 15,000 randomly sub-sampled total events and mapped onto a t-SNE plot using the high-dimensionality reduction algorithm viSNE (Breast Cancer). Samples from 5 healthy donors were also concatenated and mapped using viSNE (Healthy Donor). Each dot in the t-SNE bidimensional plot represents an unitary NK cell detected by FACS, and color code represents expression levels for each marker.

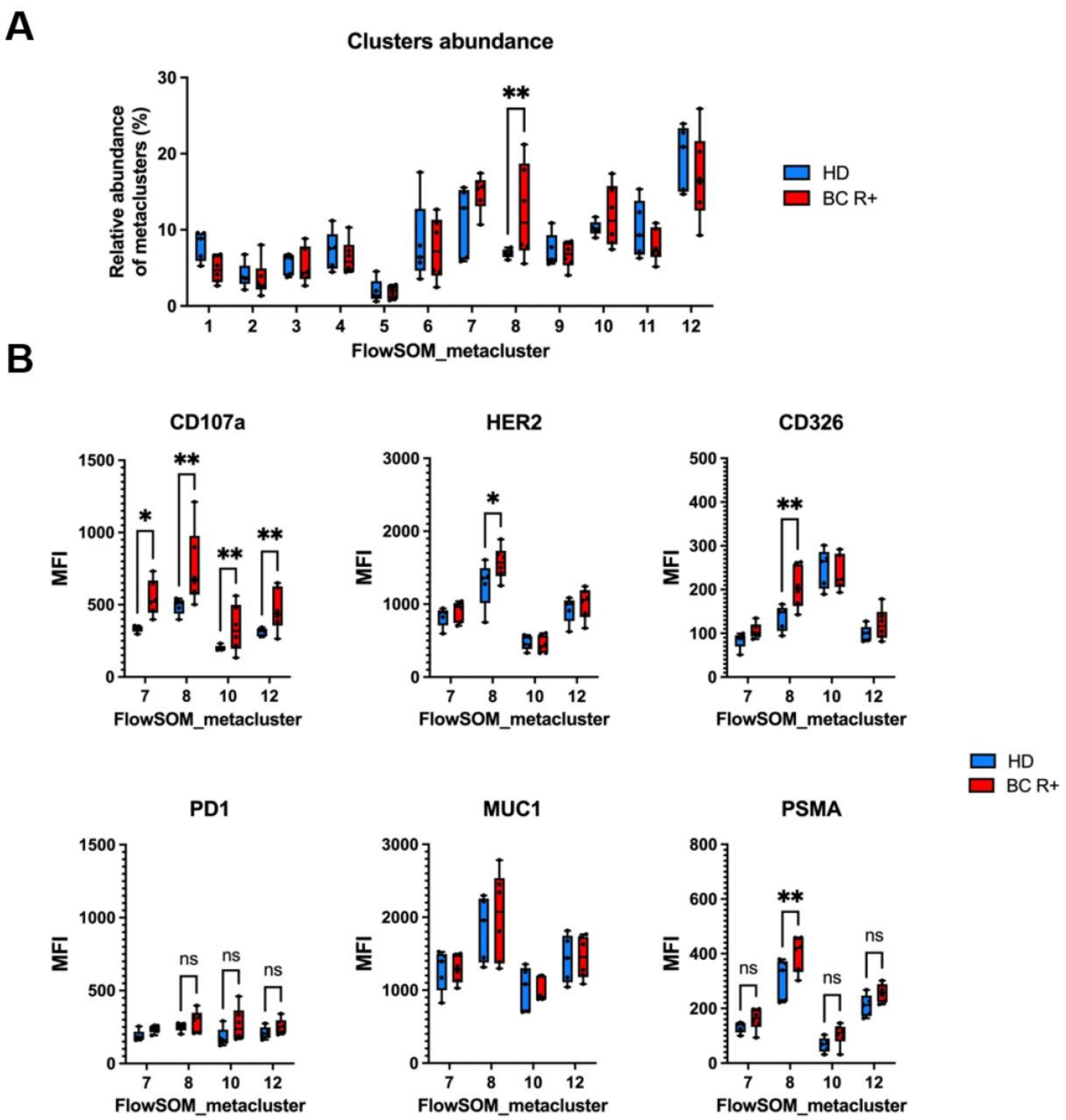

Figure S8. NK cell clusters with increased trogocytosed tumor markers are present in receptor-positive BC patients. FACS samples from healthy donors and Receptor-positive BC patients (BC R+) were analyzed as described in Figure 6. **A)** Comparison of relative frequency of each FlowSOM metacluster between HD and BC R+ patients. **B)** Median fluorescence intensity (MFI) of CD107a, PD1 and trogocytosed tumor markers expressed on CD7+CD56+ NK cells present on selected metaclusters (7, 8, 10 and 12). Graph represent box and whiskers (Min to Max), and statistical significance between HD ($n = 5$), BC R+ ($n = 6$) was determined by two-way ANOVA; * $p \leq 0.05$; ** $p \leq 0.01$.

FlowSOM metacluster ID	Breast Cancer (n=10)						Control (n=5)		*p* value	Primary marker expression phenotype on NK cells								
	All patients (n=10)		Receptor low patients (n=4)		Receptor high patients (n=6)													
	Mean (%)	SD (%)	Mean (%)	SD (%)	Mean (%)	SD (%)	Mean (%)	SD (%)		CD56	CD16	CD45RA	CD45RO	CD107a	HER2	CD326	MUC1	PSMA
1	5.4	± 1.7	6.2	± 1.3	4.8	± 2.1	8.0	± 2.0	0.183 / 0.465 / 0.108 / 0.549	+	hi	+	-	+	+	-	-	+
2	5.0	± 4.6	7.2	± 6.5	3.5	± 5.4	4.0	± 1.7	0.613 / 0.185 / 0.809 / 0.129	+	hi	+	-	-	-	+	-	-
3	7.3	± 5.6	10.4	± 8.0	5.3	± 6.6	5.5	± 1.4	0.363 / **0.044** / 0.889 / **0.034**	+	hi	+	-	-	-	+	-	-
4	7.3	± 2.2	8.7	± 1.4	6.4	± 2.3	7.2	± 2.6	0.976 / 0.543 / 0.659 / 0.334	+	hi	+	-	+	+	-	-	-
5	2.2	± 1.4	2.7	± 2.1	1.9	± 1.6	2.0	± 1.5	0.894 / 0.741 / 0.963 / 0.714	hi	-	-	-	-	+	-	-	-
6	6.9	± 3.8	6.2	± 3.6	7.5	± 3.2	8.2	± 5.4	0.507 / 0.384 / 0.694 / 0.586	hi	-	-	+	-	+	-	+	-
7	13.6	± 3.8	11.6	± 5.1	14.9	± 4.7	11.1	± 4.7	0.207 / 0.840 / 0.055 / 0.168	+	hi	+	+	hi	+	-	+	-
8	10.7	± 5.3	8.3	± 1.9	12.4	± 5.6	6.9	± 0.6	**0.049** / 0.567 / **0.006** / 0.084	+	hi	+	+	hi	+	+	+	+
9	6.4	± 2.2	6.0	± 3.1	6.7	± 2.6	7.2	± 2.2	0.672 / 0.605 / 0.778 / 0.777	+	hi	-	-	-	+	+	+	-
10	11.9	± 3.7	12.0	± 3.9	11.8	± 2.8	10.2	± 1.0	0.388 / 0.443 / 0.420 / 0.918	+	hi	+	-	+	-	+	+	-
11	7.9	± 2.2	8.0	± 2.6	7.9	± 3.1	10.1	± 3.7	0.273 / 0.271 / 0.271 / 0.977	+	hi	+	-	+	-	+	+	-
12	15.5	± 5.2	13.2	± 4.0	17.0	± 5.9	19.5	± 4.3	**0.040** / **0.010** / 0.198 / 0.119	+	hi	+	-	hi	+	-	+	+
List of abbreviations: SD, standard deviation; hi = high; NK = natural killer.																		

Table S1. FlowSOM metaclusters analysis. Frequency (mean ± SD) of metaclusters identified by FlowSOM. * P-values from ordinary two-way ANOVA (Breast tumor patients vs. Control / Patients Receptor-low vs. Control / Patients Receptor -high vs. Control / Patients Receptor -low vs. Patients Receptor -high). Values in bold correspond to $p < 0.05$.
